# Fitness of ALS-Inhibitors Herbicide Resistant Population of Loose Silky Bentgrass (*Apera spica-venti*)

**DOI:** 10.3389/fpls.2017.01660

**Published:** 2017-09-25

**Authors:** Marielle Babineau, Solvejg K. Mathiassen, Michael Kristensen, Per Kudsk

**Affiliations:** Department of Agroecology, Aarhus University Aarhus, Denmark

**Keywords:** fitness, herbicide resistance, *Apera spica-venti*, ALS inhibitor herbicide, non-target site resistance, competition

## Abstract

Herbicide resistance is an example of plant evolution caused by an increased reliance on herbicides with few sites of action to manage weed populations. This micro-evolutionary process depends on fitness, therefore the assessment of fitness differences between susceptible and resistant populations are pivotal to establish management strategies. Loose silky bentgrass (*Apera spica-venti*) is a serious weed in Eastern, Northern, and Central Europe with an increasing number of herbicide resistant populations. This study examined the fitness and growth characteristics of an ALS resistant biotype. Fitness and growth characteristics were estimated by comparing seed germination, biomass, seed yield and time to key growth stages at four crop densities of winter wheat (0, 48, 96, and 192 plants m^-2^) in a target-neighborhood design. The resistant population germinated 9–20 growing degree days (GDD) earlier than the susceptible population at 10, 16, and 22°C. No differences were observed between resistant and susceptible populations in tiller number, biomass, time to stem elongation, time to first visible inflorescence and seed production. The resistant population reached the inflorescence emergence and flowering stages in less time by 383 and 196 GDD, respectively, at a crop density of 96 winter wheat plants m^-2^ with no differences registered at other densities. This study did not observe a fitness cost to herbicide resistance, as often hypothesized. Inversely, a correlation between non-target site resistance (NTSR), earlier germination and earlier flowering time which could be interpreted as fitness benefits as these plant characteristics could be exploited by modifying the timing and site of action of herbicide application to better control ALS NTSR populations of *A. spica-venti*.

## Introduction

Herbicide resistance is an large global problem observed in 250 weed species for 23 of the 26 known herbicide sites of action (SoA) (Heap, 2016). The overuse over many generations of herbicides targeting a few specific SoA in weeds has led to the evolution of weed populations surviving increasing doses of herbicides. Evolution through natural selection in plants is a continuous process which allows weed populations to eventually overcome most selection pressures that humans apply as eradication strategies. Therefore, the use of herbicides with novel SoA might control herbicide resistant weeds for a while, but inevitably some populations will evolve resistance. The herbicide resistance mechanisms are an important factor in the strength and speed at which herbicide resistance can evolve *de novo* or spread from neighboring populations. Non-target site resistance (NTSR) implies to repurpose pre-existent stress and defense enzyme pathways to defend the plant against herbicides. NTSR pre-dates herbicide use and implicate a wide diversity of pathways and is inherited in complex manners due to polygenic identity (Délye et al., 2011; Délye, 2013). NTSR normally confers multiple resistance to other herbicide SoA (Burnet et al., 1994; Petit et al., 2009; Cummins et al., 2013; Délye, 2013). Some weeds have shown to be resistant to herbicides that they had never been exposed to Espeby et al. (2011). Therefore, management of herbicide resistance cannot rely solely on chemical solutions and must incorporate evolutionary biology knowledge when dealing with NTSR resistant weeds (Neve et al., 2009, 2014).

Traditionally, fitness is defined as the number of viable and fertile offspring contributing to the next generation. In plants, this implies that fitness could only be measured by evaluating the quantity and quality of seeds (Vila-Aiub et al., 2009). However, the number of seeds produced can depend on the health and growth of the individual. This is based on the allocation of resource theory stating that individual’s metabolism has a limiting amount of energy to allocate to vegetative growth versus reproduction and that any extra energy allocated to health (for example if plants live in a sub-optimum environment) will have a negative effect on its reproductive ability (Maxwell et al., 1990; Herms and Mattson, 1992; Park et al., 2004; Vila-Aiub et al., 2015). Therefore, fitness can also be evaluated by using proxies such as growth and health measurements. Specifically regarding agricultural weeds, fitness has been defined as survival and reproductive success in field conditions (Menchari et al., 2007) which is directly linked to competitive ability. Therefore, evaluating traits linked to competitive ability such as germination rate, vegetative growth characteristics and time needed to reach specific growth stages can indicate relative fitness differences.

The direction of natural selection depends on the selective pressure (e.g., herbicide) and also on the fitness of individuals facing this new pressure. In weeds, it was expected, and has also been observed, that herbicide resistant individuals had a lower fitness than their susceptible counterparts in the absence of the herbicide in terms of biomass and/or seed production (Bergelson and Purrington, 1996; Purrington, 2000; Ashigh and Tardif, 2009). The presence of a fitness cost could dictate different management strategies in order to reduce the resistant population. A resistant weed population with a strong fitness penalty could, in theory, be reverted to a susceptible status if the herbicide is not used.

Different resistance mechanisms [target-site (TSR) vs. NTSR] result in different fitness (Roux et al., 2005; Vila-Aiub et al., 2009; Wang et al., 2010). NTSR mechanisms are hypothesized to have a fitness cost because of negative consequences due to the changes in pathway dynamics which could even alter ecological interactions. Also negative effects on energetic resources being diverted from growth and reproduction to defense mechanism have been suggested (Jasieniuk et al., 1996; Vila-Aiub et al., 2009). Increased detoxification due to cytochrome P450 monooxygenase (P450s) in an acetyl-CoA carboxylase inhibitor (ACCase) NTSR resistant *Lolium rigidum* was correlated with a reduced seed production, biomass and growth rate (Ashigh and Tardif, 2009). Similarly, NTSR resistant *Bromus tectorum* had a reduced biomass, seed number and leaf area (Park and Mallory-Smith, 2005). However, the assumption of negative fitness in resistant weeds has been challenged as more observations of neutral and even positive fitness have arised. Neutral fitness has been observed in glyphosate NTSR resistant *Amaranthus powellii* (Giacomini et al., 2014). Positive fitness was observed in ACCase resistant *Setaria viridis* and triazine resistant *Phalaris paradoxa* (Schönfeld et al., 1987). NTSR mechanisms are diverse and a multitude of large gene families are involved such as P450s, glycosyltransferase (GT), glutathione *S*-transferase (GST), ABC-transporters, esterase, etc. (de Carvalho et al., 2009; Délye, 2013). These gene families all act in different ways (e.g., conjugation, compartmentalisation, transport) to allow the plant to survive. This multitude of factors is why the fitness of resistant weeds must be evaluated on a case by case basis (Lehnhoff et al., 2013a; Vila-Aiub et al., 2015).

Acetolactate synthase (ALS) inhibitor herbicides inhibit the synthesis of branched-chain amino acids valine, leucine and isoleucine (Duggleby et al., 2008). ALS herbicides have a high number of resistant weed species than any other SoA (Heap, 2016). Loose silky bentgrass [*Apera spica-venti* (L.) Beauv.] is one of the most serious grass weed in Northern, Eastern and Central Europe (Hamouzová et al., 2011; Massa and Gerhards, 2011; Schulz et al., 2011). At a density of 200 plants m^-2^, *A. spica-venti* reduces the yield of winter cereals up to 30%, and it can cause greater yield losses than *Alopecurus myosuroides* (Melander, 1995; Melander et al., 2008). The highest number of resistance cases in *A. spica-venti* has been reported for ALS herbicides (10 cases) followed by photosystem II (seven cases) and ACCase (three cases) (Heap, 2016). Two studies have investigated fitness differences between ALS resistant and susceptible *A. spica-venti* biotypes, both evaluating germination efficiency (Soukup et al., 2006; Gerhards and Massa, 2011). These studies revealed contradictory results where Soukup et al. (2006) found no fitness differences in a population with unknown resistance mechanism, while Gerhards and Massa (2011) found a threefold increase in germination rates in the TSR resistant biotypes. However, accurate estimation of fitness of herbicide resistant weeds is a difficult methodological task. Because of the importance of both environmental conditions and genetic background, experimental conditions and genotypes to be tested must be thoroughly controlled otherwise the fitness assessment cannot be solely attributed to the resistance allele(s) (Jasieniuk et al., 1996; Ashigh and Tardif, 2009; Vila-Aiub et al., 2009).

The lack of information of fitness in NTSR resistant *A. spica-venti* limits the implementation of evolution-knowledge-based resistance management strategies. This study aimed to evaluate the fitness and growth characteristics throughout the life cycle of ALS NTSR resistant *A. spica-venti* biotype with a randomized genetic background. The genetic backgrounds of resistant and susceptible populations were randomized over two successive generations. ALS resistance level and mechanisms were assessed. Fitness was estimated as seed germination at three different temperatures. A target-neighborhood experiment was conducted with three crop density where time to multiple key growth stages were recorded, as well as biomass and seed yield and compared to non-competitive conditions. We hypothesized that either no or higher rate of seed germination would be observed based on previous studies (Soukup et al., 2006; Massa and Gerhards, 2011) and that no differences in growth characteristics would be found based on previous field observations of ALS resistant and susceptible *A. spica-venti* populations in Denmark (Babineau, personal observation).

## Materials and Methods

### Population Selection

An ALS susceptible meta-population, named “S” was created by mixing the same proportion of seeds from five individual susceptible populations collected all over Denmark from 2004 to 2009 (**Table 1**). A meta-population approach was selected because we aimed to incorporate spatial and temporal genetic variations of susceptible *A. spica-venti* populations showing similar response to ALS. The resistant population 859P was selected because it showed a high resistance to the ALS herbicide iodosulfuron [Hussar OD, 100 g L^-1^ iodosulfuron-methyl Na + 300 g L^-1^ mefenpyr-diethyl (safener), Bayer CropScience, Germany], as well as high levels of multiple resistance to two other herbicides SoA: ACCase using fenoxaprop-P [Primera Super, 69 g L^-1^ fenoxaprop ethyl ester + 75 g L^-1^ mefenpyr-diethyle (safener), Bayer CropScience, Denmark], and fatty acid elongation using prosulfocarb (Boxer EC, 800 g L^-1^ prosulfocarb, Syngenta Crop Protection, Denmark) (**Table 1**). The resistant population was collected from a different location compared to the populations in the S meta-population (**Table 1**) which ensures different evolutionary origin (Delye et al., 2013).

**Table 1:** Selected populations of *Apera spica-venti* and respective ALS (iodosulfuron), ACCase (fenoxaprop-P) herbicide efficacy on fresh weight.

Population	Location and collection year	Iodosulfuron efficacy (dose used g a.i ha^-1^)	Fenoxaprop-P efficacy (dose used g a.i ha^-1^)
670	Dannemare, Lolland, DK 2009	98% (2.0)	100% (10.5)
672	Hadsund, Jutland, DK 2009	99% (2.0)	99% (10.5)
673	Vrå, Jutland, DK 2009	96% (2.0)	86% (10.5)
565	Hadsund, Jutland, DK 2007	83% (2.0)	94% (10.5)
482	Aabybro, Jutland, DK 2004	87% (2.0)	100% (10.5)
476	Fjenneslev, Sjealland, DK 2004	87% (2.0)	98% (10.5)
859P	Sanderumgård, Funen, DK 2011	–2% (1.5)	57% (13.8)
859F2	Flakkebjerg, DK 2015	71% (2.5)	–

### Randomization of Genetic Background

This study opted for the randomized genetic background method previously used (Roux et al., 2005; Wakelin and Preston, 2006) where a succession of two crosses (F2) was used to study the fitness of herbicide resistant in *Lolium rigidum* (Wakelin and Preston, 2006). A succession of two generations of crosses (859P × S) was performed between the resistant population and the susceptible “S” in order to obtain F2 generation populations that have a randomized genetic background. Plants from susceptible and resistant population were grown in 2 L pots (1 plant per pot) filled with a potting mixture consisting of soil, peat, and sand (2.1:1 w/w) containing all necessary micro and macro nutrients until early flowering stage. Two seed-proof isolation cabinets with automatic watering were used for cross pollination with two resistant and two susceptible plants in each cabinet. Seeds were collected from the resistant plants, threshed, cleaned, and pooled. Seeds from the F1 cross (859F1) were sown in the same conditions as the previous year for the second crossing (859F1 × S). Seeds from the randomized genetic background F2 generation (859F2) were harvested, cleaned, and kept at 4°C for at least 3 weeks to reduce primary dormancy.

### Confirmation of ALS Resistance Mechanisms

To confirm that NTSR was the mechanism causing resistance in population 859F2 and that NTSR alleles were present in the susceptible population, the frequency of six known TSR mutations in ALS were screened (Ala122, Pro197, Ala205, Asp376, Arg377, and Trp574) (Tranel et al., 2016). A total of 15 and 5 individuals were screened for 859F2 and S respectively. Oven-dried leaves (0.5 g) were grounded (2010 Geno/Grinder) for 45 s (1500 Hz) and soaked in liquid nitrogen. DNA was extracted using DNEasy Plant mini Kit (Qiagen, Germany) following manufacturer’s protocol and DNA quantity was assessed by spectrophotometer (NanoDrop-1000 v.3.1.0). PCR was performed in a total volume of 25 μL with PCR reaction buffer (10%, Thermo Fisher Scientific, Denmark), MgCl_2_ (2.5 mmol L^-1^, Thermo Fisher Scientific, Denmark), dNTPs (200 mmol L^-1^), primers (0.4 mmol L^-1^), *Taq* polymerase (2 units), and genomic DNA (200 ng). The ALS gene was sequenced in three fragments which were sequenced using the forward amplification primer (Eurofins Operon MGW, Germany). Sequences were trimmed and quality checked by eye using UGene (version 1.17.0) then aligned against the *A. spica-venti* ALS reference sequence JN646110 (Hamouzová et al., 2013) using BioEdit (version 7.2.3: Hall 1999).

To further confirm NTSR mechanisms in population 859F2, increased cytochrome P450s monooxygenase detoxification was assessed by exposing plants of each populations to malathion immediately before spraying ALS herbicides (Christopher et al., 1994). Malathion is an insecticide and inhibits some P450s in plants. Four plastic racks containing 100 plants were prepared for population 859F2, S and ALS NTSR resistant ID80. One rack remained untreated, one sprayed with iodosulfuron only, one sprayed with malathion only and the fourth with malathion prior to iodosulfuron. At the 2–3 leaf stages, two racks were sprayed with 1000 g of malathion/ha (440 g L^-1^ malathion, Cheminova). Immediately after, respective racks were sprayed with 3 g iodosulfuron/ha. Plant survival was recorded 21 days after spraying.

### ALS Resistance Bioassays on F2 Populations

The ALS resistance level of the F2 population was assessed with a discriminating dose of 2.5 g iodosulfuron ha^-1^. A total of 100 plants were sown in plastic racks filled with potting mixture. Plants were sprayed at the 2–3 leaf stage (BBCH 12–13) in a cabinet sprayer with a boom fitted with two Hardi ISO-F-020-nozzles operated at a pressure of 300 kPa, and a speed of 5.1 km h^-1^ resulting in a spray volume of 150 L ha^-1^. Fresh weight (FW) and the number of surviving plants was measured.

### Seed Germination

Seeds from 859F2 and S were cleaned using a seed blower (New Brunswick General Sheet Metal Work, New Brunswick, NJ, United States) for 1 min at an upper air velocity of 4.0 ms^-1^ to eliminate empty seeds. A 100 seeds were placed in Petri dishes (9 cm) containing four cellulose filter paper (Whatman No. 1) covered by one glass-fiber filter paper (Whatman GF/A). Five Petri dishes were prepared for each population at each of the three temperature tested: 10/6, 16/10, and 22/10°C all at 14/10 h of day/night photoperiod. Three temperatures were selected to assess germination at different conditions. The 16/10 and 22/10°C temperatures were selected following previous germination experiment with *A. spica-venti* (Andersson and Åkerblom Espeby, 2009). Seeds were imbibed with potassium nitrate (0.3%) and placed in climate cabinets for 20 days. Distilled water was added to Petri dishes when needed. Seeds were considered to have germinated when the radicle had emerged (BBCH 05). Germinated seeds were counted daily and removed. This experiment was replicated twice.

### Target-Neighborhood Experiment

A target-neighborhood design was used to compare the vegetative and reproductive ability of ALS a susceptible meta-population and the resistant biotype 859F2, in response to increasing densities of neighboring winter wheat (cv. Torp) throughout the life cycle of *A. spica-venti*. Similar target-neighborhood methods has been previously employed to study weed competition (Wakelin and Preston, 2006; Walsh et al., 2009; Keshtkar, 2015). Four winter wheat densities of 0, 48, 96, and 192 plants m^-2^ were established by sowing 0, 2, 4, and 8 plants in 10 L pots filled with potting mixture. Winter wheat plants were planted in a circular pattern order to have an equal distance among each winter wheat plant and with the *A. spica-venti* individual in the middle. The experiments were conducted from December 2015 to July 2016. Six pots per density per *A. spica-venti* population were placed on tables with an automatic watering system in greenhouse condition simulating field conditions (light and temperature) typical of the time of the year (January: 10 h light with 9.5°C in the day, June: 17 h light with 21.4°C in the day). Additional winter wheat and *A. spica-venti* plants were sown and used to replace plants that did not germinate or emerged later than the rest of the plants in their pots. Replacement was performed until the 2-leaf stage, after which no replacements were performed. This experiment was replicated twice.

A series of vegetative growth stages were measured every week for the *A. spica-venti* plants in each pot; number of tillers (BBCH 21–29), time to the beginning of stem elongation (STEM, BBCH 30), time to the first visible inflorescence (INVIS, BBCH 51), time to the first inflorescence fully emerged (INFEM, BBCH 59), time to 100% flowering (FLO) and time to 50% mature seeds (SEED50). Additionally, the above-ground biomass was measured 6 weeks after sowing using three of the six pots per density per population, and final above-ground biomass (34 weeks after sowing) on the remaining three pots. Fresh (FW) and dry weight (DW) of the *A. spica-venti* plant and winter wheat plants were measured. Reproductive ability was evaluated indirectly using the potential seed production measurement (Melander, 1995). First a seed number to panicle length ratio was calculated using 10 panicles from all populations. Then, at the final harvest, each panicle length was measured and potential seed production estimated. Aphids and powdery mildew were controlled with the insecticide imidacloprid (Confidor 70 WG, 700 g kg^-1^, Bayer CropScience, Denmark) and the fungicide metrafenon (Flexity, 300 g L^-1^, BASF A/S, Denmark) respectively. At the beginning of the flowering stage, each population was isolated so that crossing occurred only within populations.

### Statistical Analysis

Seed germination data was analyzed using a three-parameter log-logistic (LL.3) model (Eq. 1) as a function of the temperature sum according to the time-to-event approach (Ritz et al., 2013) where E is the number of germinated seeds at temperature sum x (°C), *e* (ED_50_) is the temperature sum required to reach 50% of the maximum germination, and b is the relative slope at *e* indicating germination rate.

(1) $$\begin{array}{c} E(x)=\frac{d}{1+\exp\{(b(\log(x)-\log(e))\}} \end{array}$$

Each temperature was fitted individually and then as a combined dataset. The cumulative temperature was from time of imbibition until the end of the experiment. The accumulation of thermal time (GDD, °Cd) was calculated using a base temperature of 0 (Scherner et al., 2016). The five vegetative growth stages (STE, INVIS, INFEM, FLO, and SEED50) were analyzed using a two-parameter log-logistic (LL.2) model (Eq. 2) also as a time-to-event approach where E is the number of *A. spica-venti* plants that have achieved the specific stage at temperature sum x (°Cd), with *e* being the temperature required for 50% of plants population to reach a particular growth stage.

(2) $$\begin{array}{c} E(x)=1+\exp\{(b(\log(x)-\log(e))\} \end{array}$$

Each stage was fitted separated by crop density (D0, D2, D4, and D8). The temperature sum of each week was calculated by summing the average temperature of each day of the week using the temperature log in the greenhouse. The FW was used to analyze the biomass response from the two harvest time points to the four crop densities using a density-response LL.3 (Eq. 1). In the case of biomass, *e* is the density producing a fresh weight response half way between the upper limit, *d*, and the lower limit, 0, and *b* is the slope at *e*. Seed production potential was analyzed using a LL.3 (Eq. 1) model where *e* is the density producing a seed yield half way between the upper limit, *d*, and the lower limit, 0, and b is the slope at *e*. The density-response model is exactly the same as the dose-response model (Ritz and Streibig, 2005) but substitutes herbicide dose with crop density. All time-to-event and density-response model fittings were followed by graphical analysis of the distribution of residuals and a test for lack of fit comparing the residual sum of square of a two-way analysis of variance and the non-linear regression. If the graphical analysis of the residuals was not satisfactory, the biomass was transformed using a Box-Cox transformation and the model was fitted again. Once each population was fitted with a satisfactory model, the ED_50_ was compared between populations using the Bonferroni correction (Benjamini and Hochberg, 1995), an adjustment for multiple comparisons. Time-to-event and dose-response analysis were all performed in R (R Core Team, 2015) using package drc (Ritz and Streibig, 2005).

The tiller number values were analyzed in R (R Core Team, 2015) using linear regression with an alpha significance threshold of 95%. An ANOVA was fitted to the regression, followed by a pairwise *t*-test with a Bonferroni correction for multiple *p*-value comparisons. Finally, a Tukey honest significance differences test was performed to identify which populations were significantly different. Tiller numbers were analyzed separately by week, by crop density and all densities together.

## Results

### ALS Resistance Level and Mechanisms

**FIGURE 1:**
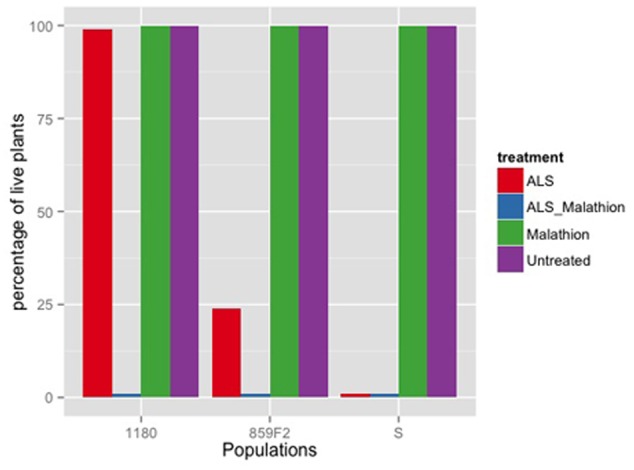
Relative number of alive plants for ALS NTSR population 859F2 and susceptible population of *Apera spica-venti* treated with 3 g of iodosulfuron, 1000 g of malathione and both treatments.

The 859F2 population with a randomized genetic background was less resistant than the parental 859P populations (**Table 1**). The F2 population was nonetheless showing a low level of resistance to iodosulfuron (**Table 1**). The NTSR experiment using malathione (**Figure 1**) showed that malathione alone had no effect on plant survival and that the 859F2 population had an intermediate ALS resistance level compared to S and ID80. The application of malathione prior to the ALS herbicide reduced plant survival to zero in all populations. The malathione synergy experiment together with the absence of the known mutations causing TSR and the multiple resistance status of its parental population strongly implied that resistance was due to NTSR. The randomization of the genetic background therefore decreased the resistance levels in the F2 populations, but did not eliminate the resistance alleles.

### Germination

**FIGURE 2:**
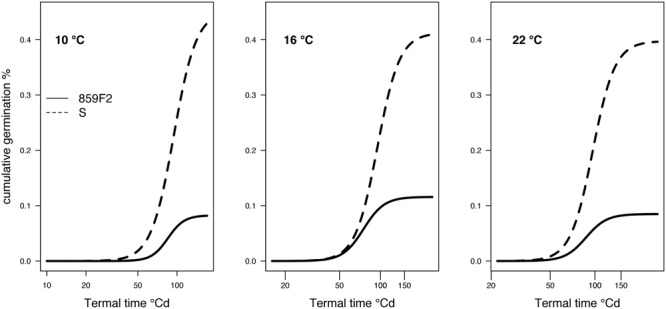
Germination results for ALS herbicide NTSR resistant (R) and susceptible (S) populations of *A. spica-venti* tested at three temperatures.

The ALS resistant population showed significant differences in the maximum germination (*d*) and ED50 (*e*) for seed germination at all temperatures compared to the S meta-population (**Figure 2** and **Table 2**). 859F2 showed significant difference in germination rate (*b*) only at 10°C. The difference in the ED_50_ °Cd between susceptible and resistant biotypes was largest when germination took place at 16°C (19.8 °Cd) and similar (9.5 °Cd) at 22 and 10°C (**Table 2**). The susceptible population produced a higher number of germinating seeds than the resistant population (**Figure 2**).

**Table 2:** Parameter values with standard error (st) for the germination experiment performed at 10, 16, and 22°C for the four populations of *A. spica-venti* fitted with a LL.3 time-to-event model.

Temperature (°C)	Population	Germination rate- b (st)	Maximal germination -d (st)	ED50 – e (st) °Cd
10	R	**–6.9 (0.7)**	**0.08 (0.008)**	**85.0 (2.3)**
	S	**–**4.9 (0.2)	0.4 (0.01)	94.2 (1.8)
16	R	**–**5.7 (0.4)	**0.1 (0.01)**	**75.1 (2.1)**
	S	**–**5.3 (0.2)	0.4 (0.01)	94.9 (1.5)
22	R	**–**6.2 (0.6)	**0.08 (0.008)**	**87.0 (2.6)**
	S	**–**5.8 (0.2)	0.3 (0.01)	96.5 (1.4)

*Bold values indicate significant difference (*p*-value < 0.05) between the ALS susceptible (S) population and resistant (R) population.*

### Vegetative and Reproductive Growth Stages

**FIGURE 3:**
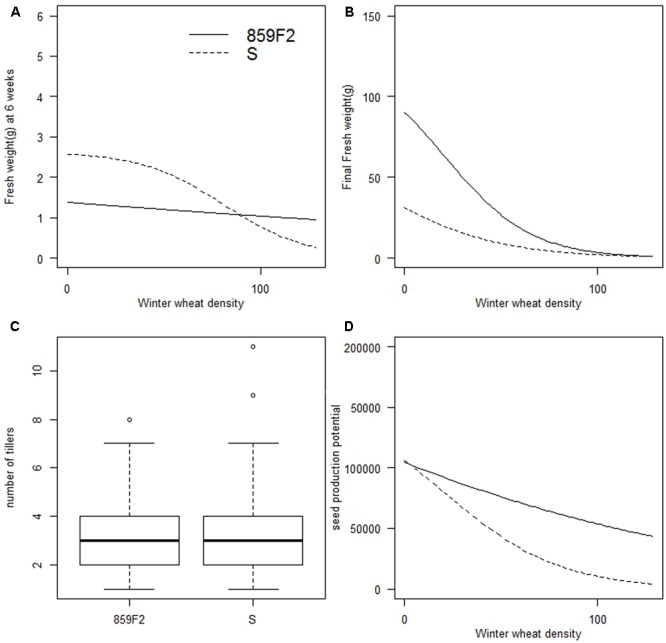
Vegetative and reproductive traits showing no significant differences between ALS susceptible and resistant populations of *A. spica-venti*. **(A)** Density-response curves for biomass 6 weeks after sowing. **(B)** Density-response curves for final biomass. **(C)** Tiller number at week 11 with four crop densities combined. **(D)** Density-response curves for potential seed production at competitive density of 96 winter wheat plant m^-2^ (D4).

No significant differences were found for the number of tillers between susceptible and resistant populations for the four time points recorded (**Figure 3C**). No significant differences in *e* were found for biomass 6 weeks after sowing and final biomass between populations (**Figures 3A,B** and Supplementary Table S1). Further, no significant differences were observed in panicle number or potential seed production between populations (**Figure 3D**). The *R*^2^ value for seed number as a function of panicle length was 0.71 which is in line with similar estimates also made on *A. spica-venti* panicles (Melander, 1995). Across all four crop densities, population 859F2 and S produced a mean of 123107 (± 164178) and 81433 (± 99354) seeds respectively, showing large variation between densities but no significant differences between the two populations. Tiller number, biomass, panicle number, and seed production were all inversely proportional to crop density in all populations.

**FIGURE 4:**
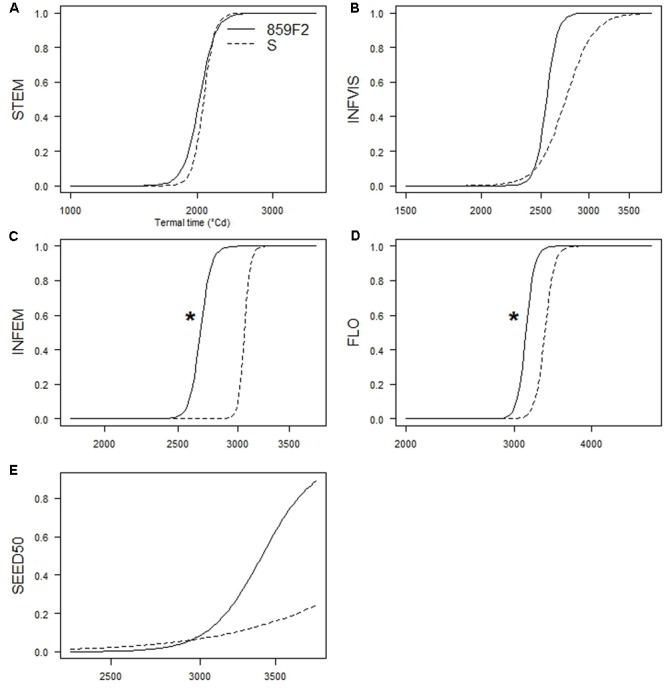
Time-to-event relative curves for the five growth stages measured in *A. spica-venti* at density 4. **(A)** Beginning of stem elongation (STEM). **(B)** First visible inflorescence (INVIS). **(C)** First emerged inflorescence (INFEM). **(D)** 100% flowering (FLO). **(E)** 50% mature seed production (SEED50). Significant pair differences indicated with an asterisk.

**FIGURE 5:**
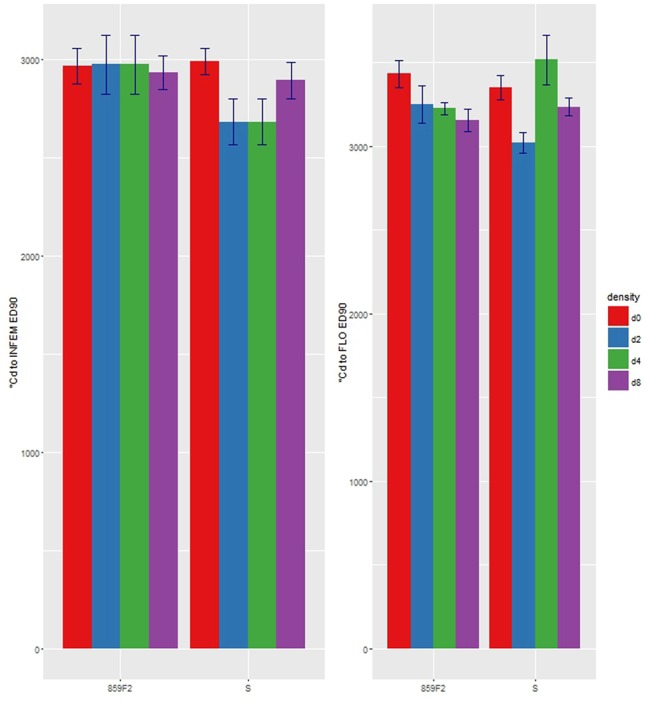
ED90 with standard error of first emerged inflorescence (INFEM) and 100% flowering (FLO) growth stages by crop density for ALS resistant (859F2) and susceptible (S) populations of *A. spica-venti*.

There were no significant differences in *e* for STEM, INVIS, or SEED50 (**Figures 4A,B,E**) at any crop density. It took on average 1 800 °Cd and 2 500 °Cd respectively to reach STEM and INFVIS by both resistant and susceptible populations (**Table 3** and Supplementary Table S2). However, significant differences in ED_50_ were observed at INFEM and FLO stages at D4 (**Table 3** and **Figure 4**). At the INFEM stage at D4, 859F2 was faster by 383 °Cd, for FLO at D4, 859F2 was faster by 196 °Cd (**Table 3**). No significant differences were observed at D0, D2, or D8 in any of the five growth stages (**Figure 4** and Supplementary Table S2). Even in stages with no significant differences (STEM, INFVIS, SEED50) trends between the different densities and populations can be seen (**Figure 4**). The differences between resistant and susceptible population were also observable when estimating the GDD until 90% of the population reached INFEM and FLO (**Figure 5**).

**Table 3:** Parameter values from the LL.2 model fitted to five growth stages at crop density D4, 96 winter wheat by m**^-^**^2^.

Growth stage	Population	D4	
		*b*	*e*
STEM	R	**–**23.1 (8)	2018 (65)
	S	**–**33.7 (17)	2064 (67)
INFVIS	R	**–**47 (18)	2555 (42)
	S	**–**19 (10)	2748 (189)
INFEM	R	**–**20 (6)	**2672 (97) *p*-value 0.011**
	S	**–**36 (19)	2526 (75)
FLO	R	**–**106 (53)	**3162 (31) *p*-value 0.001**
	S	**–**47 (31)	3358 (94)
SEED	R	**–**34 (15)	3456 (78)
	S	**–**7 (5)	4289 (754)

*Significant differences between ALS susceptible and resistant population indicated by bold numbers. Growth stage abbreviations as follows: STEM, beginning of stem elongation; INFVIS, first inflorescence visible; INFEM, first inflorescence emerged; FLO, 100% flowering; SEED50, 50% mature seed.*

## Discussion

The fitness and competitive ability of one ALS NTSR resistant population of *A. spica-venti* was tested against a susceptible biotype throughout its life cycle. No differences were found in terms of tiller number, biomass, time to stem elongation, time to first visible inflorescence, time to 50% mature seed, nor in final yield. The absence of biomass differences in competitive conditions was also observed in NTSR resistant *Avena fatua* (Lehnhoff et al., 2013a). A significant correlation was observed for the resistant population in terms of seed germination, time to emerged inflorescence (INFEM), and time to 100% flowering (FLO).

The resistant population germinated 9 to 20 °Cd earlier than the susceptible population which in Denmark would represent 1–2 days difference at the time of sowing of winter cereals. This result is consistent with the results reported for TSR ALS resistant *B. tectorum* and *Kochia scoparia* biotypes which showed significantly higher germination rate at low temperatures (5°C) compared to the corresponding susceptible biotypes (Dyer et al., 1993; Park et al., 2004). Differences in germination rate were observed only at the lowest temperature unlike a previous study of ALS resistant *A. spica-venti* where a threefold increase in germination for the TSR (Pro197, Trp574, and Arg377) resistant biotypes was observed (Gerhards and Massa, 2011; Massa et al., 2011) at 20°C/15°C with 12 h photoperiod. This difference could be due to resistance mechanisms as TSR and NTSR have very different genetic implications and different genes are modified to allow survival (Délye, 2013). Single nucleotide mutations are hypothesized to have negative pleiotropic effect on gene function whereas NTSR mechanisms are thought to have negative effect on growth as a limited amount of resources have to be allocated to either defense or growth (Vila-Aiub et al., 2015). Therefore, the modified ALS gene, which is expressed in seeds and is essential in the synthesis of amino acids leucine, valine and isoleucine, in the TSR individuals could explain the increased germination rate found by Gerhards and Massa (2011). A reduction in total germination for the resistant population was observed in this study compared to the susceptible, in contrast to Soukup et al. (2006) who found no differences between four ALS resistant and four susceptible field populations of *A. spica-venti* tested in Petri dishes (20°C) and pot experiment. The difference in total germination could be due to differences in dormancy proportions between resistant and susceptible, as *A. spica-venti* has previously shown variation in seed primary dormancy (Andersson and Åkerblom Espeby, 2009).

Weeds that germinate earlier can better compete with crop and other weeds which gives them a considerable advantage (Kleemann and Gill, 2013; Owen et al., 2015). More than 30 different P450s are expressed in *A. spica-venti* seeds at different germination stages and NTSR, via an increased cytochrome P450s activity, can be hypothesized to have a pleiotropic effect on germination (Babineau et al., 2017). The results of this study also show differences in the magnitude with germination at 16°C showing the largest difference between resistant and susceptible population compared to 10 and 22°C. Fitness differences between germination temperatures were also observed in resistant *B. tectorum* showing germination differences between ALS resistant and susceptible populations only at low (5°C vs. 15°C and 25°C) temperature (Park et al., 2004). This could be explained by the known association of germination efficiency and temperature and might reflect regional adaption. Alternatively, there could be linkage between genes associated with germination timing and P450s under selection for ALS resistance. This is supported by the fact that P450s genes in *Arabidopsis thaliana* have been shown to evolved from ancient whole genome and successive gene duplication of P450s tandem arrays resulting in P40s genes being tightly linked and distributed on multiple chromosomes (Bak et al., 2011). Several P450s (CYP707A1-A4) have been found to be directly involved in decreasing the levels of abscisic acid (ABA) during seed imbibition in *A. thaliana* which allows seeds to germinate by lowering dormancy (Schopfer et al., 1979; Kushiro et al., 2004). The same relationship between P450s and seed germination has not yet been established in *A. spica-venti*, but the increased germination in individuals showing a high variation in P450s due to herbicide selection this is possible.

At the medium density of 96 wheat plants m^-2^ the ALS NTSR resistant population took less time to reach INFEM and FLO by 383 and 196 °Cd respectively, which corresponds to around 25 and 13 days, respectively, in field condition in Denmark. The importance of timing of trait has been underlined before (Paris et al., 2008). Similarly, NTSR resistant *A. fatua* reached anthesis earlier than susceptible biotypes (Lehnhoff et al., 2013b). Flowering time appears to be a plastic trait as flowering time in *Raphanus raphanistrum* was halved within five generations using directional selection (Ashworth et al., 2016). The ability to reach inflorescence emergence and flowering earlier is an advantage to weed populations allowing them to escape potential eradication by late season management strategies or harvesting. Herbicide NTSR resistance mechanisms can have different pleiotropic effects that could manifest themselves only at specific growth stages. In the field, difference in flowering time could imply selective interbreeding only between members of a resistant population which could result in sub-population structuring and differentiation over consecutive generations. Therefore, different ALS resistance mechanisms present in neighboring populations would not mix due to gene flow and populations could instead become increasingly genetically isolated.

The effect of crop competition on fitness and growth characteristics of ALS resistant biotypes shows an interesting pattern in this study. There were no significant differences at low densities (D0 and D2) neither at the high density (D8) in any of the five growth stages. Growth differences only manifested at 96 winter wheat plants m^-2^ (D4). This result indicates that high crop density reduces the fitness of resistant biotypes to an equal level to the fitness of susceptible populations as previous studies have been shown (Park et al., 2004; Paris et al., 2008). Recovery of growth timing differences at intermediate crop density and not at the lower densities has also been shown previously at D4 in *A. fatua* (Lehnhoff et al., 2013a,b). This could indicate differences in the regulation of NTSR constitutively expressed genes. Competition is known to influence the traits that exhibit fitness differences. Evolution in competitive conditions will favor fitness cost on early growth traits while evolution in non-competitive conditions will favor fitness cost on later growth traits (Paris et al., 2008). However, a fitness advantage of the resistant population in both early (germination) and later (inflorescence emergence and flowering) growth stages might imply very different and variable competitive conditions when ALS resistance evolved. The crop density in which we observe significant growth differences is much lower than the realistic wheat density (200–300 m^-2^) in farm conditions. We did not observed any differences in the growth of NTSR *A. spica-venti* at the lower end of the farm wheat density spectrum (D8; 192 m^-2^). Therefore, the timing of growth stages differences observed in this study most likely will not be observed in field conditions at normal wheat densities. However, in crops with lower farm densities or different competitive ability compared to wheat, the differences observed here in the timing of growth stages for this weed could be observable and would be worth investigating.

This study did not demonstrated a fitness cost of resistance, as often hypothesized, but found a correlation with earlier germination and growth which could be a fitness benefit in some field conditions. Earlier germination can translate to a better access to nitrogen and nutrients and early vigor. However, this benefit could turn into a cost in case of false seedbed or pre-emergence application of foliar-active herbicides.

Fitness benefits have been attributed to two possible processes: compensatory and replacement hypothesis (McKenzie et al., 1982; Andersson, 2003; Paris et al., 2008; Darmency et al., 2015). Compensatory mechanism implies specific pleiotropic consequences, where the resistance allele is linked to other alleles (modifier genes) that help compensate for the fitness cost of resistance (Andersson, 2003; Darmency et al., 2015). These modifier genes can be linked to resistance alleles from the beginning or can evolve rapidly (McKenzie et al., 1982). The replacement hypothesis argues that resistance alleles carrying a fitness penalty can be replaced over time with similar resistance alleles that do not carry a fitness penalty (Paris et al., 2008). In the case of the resistant population examined in this study, both hypotheses could apply since it is unknown how long ago the resistance evolved and therefore replacement or modifier genes could have had enough time to evolve and be fixed.

In general, fitness is defined as “to survive and produce a number of fertile and viable offspring that will contribute to the next generation” (Barker, 2009). In strict evolutionary terms, there were no fitness cost or benefit observed in this study as the final seed number (viability was not measured) was not different between resistant and susceptible *A. spica-venti*. However, in an agricultural landscape, plants (crops and weeds) are managed throughout their life cycle and their competitive ability has a direct effect on their survival which often cannot be measured solely based on seed production. Small differences in competitive ability or in management practices can have large effect on weed population level which will obviously be observed in seed yield at the end of the season. But this difference in seed yield will not identify the precise life stage and precise differences that led to the higher total fitness which is key in weed management. Therefore fitness evaluation and measurement in agricultural conditions has to be enlarged to encompass growth and competitive differences (Vila-Aiub et al., 2009, 2015) much more prominently than viable offspring evaluation seen in natural environmental studies (Leimu et al., 2006). For these reasons, we surmise that fitness cost or benefits in weeds should consistently aim to assess plants throughout their life cycle. The strict definition of fitness based on viable offspring number is not appropriate for evaluating plants in intensively managed anthropogenic environments.

Another theoretical aspect emerging from this study is the use of randomized genetic background in studying NTSR mechanisms. This randomization of the genetic background method used to compare individual fitness in herbicide resistant weeds was originally designed for one allele difference, e.g., TSR and was used as such (Roux et al., 2004; Menchari et al., 2007; Delye et al., 2013). The handful of studies investigating fitness cost of NTSR mechanisms have used resistant and susceptible individuals from the same population (P450s; Vila-Aiub et al., 2005), reduced translocation (Wakelin and Preston, 2006; Pedersen et al., 2007), or the genetic background randomization (EPSPS gene amplification, Giacomini et al., 2014). However, just as in our study, the exact NTSR allele(s) was not identified (e.g., which P450s isoenzyme) and fitness evaluation was based on bulk assessment of involvement of one or multiple large gene families. The problem with evaluating fitness cost from NTSR is that, as mentioned before, these mechanisms are assumed to be polygenic and generally involves multiple alleles, from multiple loci across a varied functional background. All of these have not only their own evolutionary history regarding herbicide resistance, genetic linkage and inheritance, but most likely have their own fitness cost associated. This bulk assessment of NTSR fitness cost comprises a mixture of genes that could have additive, multiplicative, agonist, antagonist, and compensatory effects on fitness phenotype depending on the combination that has evolved in a particular population in a particular weed species.

The aim of the genetic randomization method is to have similar genetic background except for the alleles conferring resistance by equally mixing the whole genetic background of both herbicide resistant and susceptible individuals by performing crosses for a few generations. In this context, it is uncertain how the various NTSR alleles will be distributed after a few generation of genetic randomization background; they could be randomly distributed but then no “true” susceptible will be found, they could be lost, or some alleles could co-segregate due to linkage or recombination. The latter would result in individuals that are resistant to herbicide due to NTSR but not the same allele combinations than found in the field which could not guide weed management strategies. The assessment of field populations displaying NTSR will be difficult because of the reasons explained above and also because NTSR mechanisms pre-exist at a certain degree in every individuals no matter their phenotype. The study of the bulk effect of NTSR mechanism on fitness is useful as it represents more closely the situation in the field and also gives a response to the question of the presence or absence of a fitness cost. In the absence of a fitness cost, the resistant individuals are not likely to decrease without the herbicide and other management strategies can be sought. If a fitness cost (or benefit) is detected from the bulk analysis, then the identification of the specific alleles and their individual effect on fitness cost becomes necessary.

An appropriate method for estimating fitness cost from individual NTSR alleles and their respective roles in fitness would be to create different lines with very similar genetic backgrounds (using near isogenic lines or model plant *A. thaliana*) but differing by only one allele. Increasing gradually the number of NTSR alleles in some lines could allow to effectively estimate the epistatic effect (cumulative, antagonist, etc.) of the different genes involved in NTSR. This method requires time, large spatial resources and genetic transformation ability (if used on a weed species) but most importantly requires to identify each NTSR alleles thought to be involved in herbicide resistance in a particular individuals showing a fitness cost which would necessitate a differential gene expression pre-analysis. This method could also allow testing the hypothesis of modifier genes. To our knowledge, no study has performed such an analysis yet but with the increasing number of herbicide NTSR fitness cost studies (Park and Mallory-Smith, 2005; Vila-Aiub et al., 2005; Pedersen et al., 2007; Preston and Wakelin, 2008; Giacomini et al., 2014) and transcriptomic studies identifying herbicide resistant genes (Hu et al., 2009; Cummins et al., 2013; Gaines et al., 2014; Duhoux et al., 2015) this method has the basic knowledge available to be used. Once used across a range of NTSR alleles, the comparison with different NTSR weed species or populations might inform about the universality of fitness cost associated to certain alleles which could help weed management. In our study for example, the inconsistent fitness difference observed (germination and flowering stage only) could be explained by the role of different NTSR genes having pleiotropic effects at different life stages.

The presence of fitness differences between herbicide resistant and susceptible biotypes has been used previously to develop management strategies that exploit those differences (Neve et al., 2009, 2014; Preston et al., 2009), however, most have focused on a fitness cost assumption. The finding of potential beneficial differences in ALS NTSR resistant *A. spica-venti*, as in this study and by Gerhards and Massa (Gerhards and Massa, 2011), calls for the development of management strategies that take into account different direction and magnitudes of fitness consequences (Colbach et al., 2006). The early germination observed for the resistant biotype in this study could imply a selective eradication by pre-emergence application of foliar-active herbicides or stale seedbed methods. The earlier flowering and seed production stages will be difficult to manage. However, flowering differences were recovered at a crop density lower than in Danish fields, which means that at the current sowing densities of winter wheat these differences are most likely non-existent and would not cause new management problems. The management of such growth differences in NTSR biotypes cannot be chemically managed because of the high risk of multiple resistance, which emphasizes the importance of herbicide resistance prevention strategies.

Lastly, the results observed here come from only one NTSR population. Therefore, the investigation of more *A. spica-venti* NTSR populations in terms of germination and growth characteristics might confirm, or nuance, the results observed here, as sample number is an important factor is the estimation of herbicide resistance fitness cost in weeds (Cousens et al., 1997).

## Conclusion

This study observed a correlation between germination and growth differences and NTSR resistance in an *A. spica-venti* population. The resistant population germinated 9–20 GDD earlier at 10, 16, and 22°C, reached inflorescence 383 GDD earlier and flowered 196 GDD earlier at a density of 96 wheat m^-2^ compared to the susceptible population. No differences were observed at other wheat densities or regarding tiller number, biomass, time to stem elongation, time to first visible inflorescence, time to 50% mature seed, nor in final yield. The differences in growth characteristics identified could be used to better manage ALS NTSR loose silky bentgrass populations in the future.

## Author Contributions

MB designed, conducted, analyzed data and drafted the manuscript. SM, MK, and PK designed the experiment, advised with data interpretation and reviewed the manuscript.

## Conflict of Interest Statement

The authors declare that the research was conducted in the absence of any commercial or financial relationships that could be construed as a potential conflict of interest.
