# Supplementary material for: Fitness of ALS-Inhibitors Herbicide Resistant Population of Loose Silky Bentgrass (Apera spica-venti)
Source: Front Plant Sci. 2017 Sep 25;8:1660. doi: 10.3389/fpls.2017.01660 (PMC5622297; doi:10.3389/fpls.2017.01660)
Supplement: Supplementary file 1 [file Table_1.DOCX]

**Supporting Material.**

Table S1. Parameter values with standard error for the LL.3 model fitted to the *A. spica-venti* fresh weight biomass 6 weeks after sowing and at seed shedding. Bold values indicate significant differences (pvalue < 0.05) between populations.

| Harvest time | Pop | *B* (slope) | *d* (g/pot) | *e* (ED50) |
| --- | --- | --- | --- | --- |
| FW after 6 weeks | R | 0.5 (0.5) | 3.1 (0.9) | 0.2 (0.7) |
|  | S | 1.3 (0.7) | 1.9 (0.6) | 2.4 (1.5) |
| Final FW | R | 1.9 (4.1) | 124.7 (10.8) | 0.6 (1.7) |
|  | S | 1.4 (10.1) | 83.9 (10.8) | 0.3 (4.2) |

Table S2. Parameter values from the LL.2 model fitted to five growth stages by crop density: D0, 0 winter wheat by m^-2^, D2, 48 winter wheat by m^-2^, and D8, 192 winter wheat by m^-2^. Significant differences between ALS susceptible and resistant population indicated by bold numbers. Growth stage abbreviations as follows: STEM, beginning of stem elongation, INFVIS, first inflorescence visible, INFEM, first inflorescence emerged, FLO, 100% flowering, SEED50, 50% mature seed.

| Growth stage | Population | D0 | | D2 | | D8 | |
| --- | --- | --- | --- | --- | --- | --- | --- |
|  |  | *b* | *e* | *b* | *e* | *b* | *e* |
| STEM | R | -26.9 (10) | 1842 (50) | -16.6 (5) | 1878 (84) | -36.0 (15) | 2238 (53) |
|  | S | -27.1 (9) | 1864 (52) | -43.3 (18) | 1931 (40) | -15.9 (5) | 2417 (114) |
| INFVIS | R | -33 (12) | 2567 (57) | -26 (8) | 2482 (65) | -40 (17) | 2596 (53) |
|  | S | -45 (18) | 2618 (44) | -30 (15) | 2364 (85) | -18 (6) | 2734 (109) |
| INFEM | R | -34 (12) | 2783 (61) | -20 (6) | 2672 (97) | -43 (18) | 2790 (55) |
|  | S | -48 (18) | 2858 (47) | -36 (19) | 2526 (75) | -34 (12) | 2714 (58) |
| FLO | R | -48 (18) | 3280 (53) | -30 (10) | 3029 (73) | -69 (34) | 3058 (41) |
|  | S | -55 (22) | 3223 (45) | -160 (118) | 2983 (54) | -85 (47) | 3056 (28) |
| SEED | R | -8 (3) | 4320 (424) | -38 (16) | 3445 (69) | -41 (21) | 3456 (83) |
|  | S | -2 (1) | 5797 (1784) | -178 (602) | 3368 (49) | -15 (14) | 3916 (516) |
